# Seasonal Distribution and Historic Trends in Abundance of White Sharks, *Carcharodon carcharias*, in the Western North Atlantic Ocean

**DOI:** 10.1371/journal.pone.0099240

**Published:** 2014-06-11

**Authors:** Tobey H. Curtis, Camilla T. McCandless, John K. Carlson, Gregory B. Skomal, Nancy E. Kohler, Lisa J. Natanson, George H. Burgess, John J. Hoey, Harold L. Pratt

**Affiliations:** 1 National Oceanic and Atmospheric Administration, National Marine Fisheries Service, Greater Atlantic Regional Fisheries Office, Gloucester, Massachusetts, United States of America; 2 National Oceanic and Atmospheric Administration, National Marine Fisheries Service, Northeast Fisheries Science Center, Narragansett, Rhode Island, United States of America; 3 National Oceanic and Atmospheric Administration, National Marine Fisheries Service, Southeast Fisheries Science Center, Panama City, Florida, United States of America; 4 Massachusetts Division of Marine Fisheries, New Bedford, Massachusetts, United States of America; 5 Florida Program for Shark Research, Florida Museum of Natural History, University of Florida, Gainesville, Florida, United States of America; 6 Mote Marine Laboratory, Summerland Key, Florida, United States of America; University of California Davis, United States of America

## Abstract

Despite recent advances in field research on white sharks (*Carcharodon carcharias*) in several regions around the world, opportunistic capture and sighting records remain the primary source of information on this species in the northwest Atlantic Ocean (NWA). Previous studies using limited datasets have suggested a precipitous decline in the abundance of white sharks from this region, but considerable uncertainty in these studies warrants additional investigation. This study builds upon previously published data combined with recent unpublished records and presents a synthesis of 649 confirmed white shark records from the NWA compiled over a 210-year period (1800-2010), resulting in the largest white shark dataset yet compiled from this region. These comprehensive records were used to update our understanding of their seasonal distribution, relative abundance trends, habitat use, and fisheries interactions. All life stages were present in continental shelf waters year-round, but median latitude of white shark occurrence varied seasonally. White sharks primarily occurred between Massachusetts and New Jersey during summer and off Florida during winter, with broad distribution along the coast during spring and fall. The majority of fishing gear interactions occurred with rod and reel, longline, and gillnet gears. Historic abundance trends from multiple sources support a significant decline in white shark abundance in the 1970s and 1980s, but there have been apparent increases in abundance since the 1990s when a variety of conservation measures were implemented. Though the white shark's inherent vulnerability to exploitation warrants continued protections, our results suggest a more optimistic outlook for the recovery of this iconic predator in the Atlantic.

## Introduction

The white shark *Carcharodon carcharias* is one of the largest, most widespread ocean predators distributed in sub-polar to tropical seas of both hemispheres [1]. White sharks are important apex predators that occupy trophic levels similar to that of carnivorous marine mammals (trophic level  =  4.5) [2]–[3]. While white shark productivity (expressed as intrinsic rates of increase or population rebound potentials) falls along the midpoint of a continuum of productivity values calculated for a suite of shark species [4]–[5], they may have naturally low abundance [6] and possess general life history traits that make them vulnerable to exploitation [7]–[9]. Although white sharks have not historically been subjected to directed fisheries, there are numerous accounts of incidental captures in commercial fisheries worldwide [1], [10]–[14]. Moreover, their iconic status and highly valued jaws and fins have subjected them to targeted recreational and trophy fisheries where or when their populations have been unprotected [1], [11].

To date, only Baum et al. [15] and McPherson and Myers [16] have attempted any quantitative assessment of the status of the white shark population in the northwest Atlantic Ocean (NWA). While some of these results have been criticized as unreliable and overly pessimistic [17], analysis of pelagic longline fishery logbook data from the NWA suggested a sharp decline (between 59 and 89%) in white shark numbers between 1986 and 2000 [15]. Similarly, using sparse sightings data (N = 31) from Atlantic Canada, McPherson and Myers [16] estimated a 3-950 fold decrease in white shark population size between 1926 and 1988. Due to studies such as these, evidence of population declines in other regions around the world (e.g., [18]–[19]), and their iconic and charismatic nature, white sharks have been afforded some of the highest level of protection of any elasmobranch. For example, they have been listed on the appendices of The United Nations Convention on Law of the Sea (UNCLOS), the Convention on International Trade in Endangered Species of Wild Fauna and Flora (CITES), and the Convention for the Conservation of Migratory Species (CMS). The World Conservation Union (IUCN) currently lists the white shark globally as ‘Vulnerable’ [20]. In the NWA, The Committee on the Status of Endangered Wildlife in Canada (COSEWIC) has recommended that white sharks be listed as “Endangered,” and they have been listed as a prohibited species (i.e., no commercial or recreational harvest) in US waters since 1997 [21]. Due to these conservation concerns, and the high uncertainty associated with previous studies [15]–[16], there is a need to better understand the historic and current status of white sharks in the NWA, incorporating as much reliable data as possible.

Despite recent advances in field research on white sharks in several regions around the world (e.g., [22]–[23]), opportunistic capture and sighting records remain the primary source of information on this species in the NWA [14], [16], [24]–[25]. This is due to their sparse distribution and a historic lack of discrete coastal aggregation sites in this region. Casey and Pratt [24] provided a qualitative assessment of the distribution of NWA white sharks, but this study took place before the significant expansion in the 1980s of directed large coastal shark fisheries in the US Atlantic (e.g., [26]–[27]). White sharks were found to range from Newfoundland, Canada to the Gulf of Mexico and northern Caribbean Sea, but were most frequently encountered from the Gulf of Maine south to Cape Hatteras, North Carolina [24]. They have been considered only occasional visitors to the warmer waters off the southeastern US and Gulf of Mexico [24], [28]–[31].

Herein, we report on the patterns of distribution and relative abundance of white sharks in the NWA region based on a comprehensive compilation of historic and recent white shark capture and sighting records. A variety of fishery-dependent and -independent sources were synthesized, resulting in the largest white shark dataset yet compiled from this region. We provide a robust description of their historical abundance trends, spatio-temporal distribution, fishery interactions, and essential habitats. This updated information will improve the conservation and management of white sharks regionally and internationally, and provide a new baseline for future studies.

## Methods

White shark occurrence records were collected from numerous sources, including landings data, commercial fishery observer programs, recreational tournament information, scientific research surveys, commercial and recreational fishermen, collaborating scientists, newspaper articles, personal communications, and the scientific literature ([14], [24] and references therein, [25], [31]–[33]). Due to species misreporting problems in the pelagic longline fishery [17], logbook records from this fishery were considered unreliable and excluded. The data in each record typically included date, location, measured or estimated shark total length (TL), and capture gear (unless a visual observation). Lengths estimated at greater than 6 m were considered unreliable [34]. Where lengths were reported in fork length, conversion to TL was performed using the formula in Kohler et al. [35]. Based on published length-at-age and length-at-maturity estimates [7]–[8], [36], sharks were classified as neonate (<1.5 m TL), young-of-the-year (YOY, <1.75 m TL), juvenile (1.75–3.79 and 1.75–4.5 m TL for males and females, respectively), or mature (>3.79 and >4.5 m TL for males and females, respectively). Some records had more complete data including shark weight, sex, stomach contents, photographs, water temperature, depth, or other observations. All records were given a subjective reliability ranking of A, B, C, or F similar to that described by Casey and Pratt [24] and Skomal et al. [14]. Records receiving a low ranking of C or F, in which the identification of the white shark seemed suspect, could not be corroborated, and/or lacked photographic evidence, were excluded from the analysis.

### Distribution analysis

All records were analyzed with reference to spatial and temporal patterns of presence, as well as bottom depth and sea surface temperature (SST), when recorded. If not reported, white shark sighting locations (latitude and longitude) were assigned where possible. Data were plotted using Geographic Information System (GIS) software (ArcGIS v. 10.0, ESRI, Redlands, California). Bottom depth was subsequently assigned to each observation by matching the position to ETOPO1 Ocean Relief Model bathymetry in ArcGIS. To investigate seasonal changes in distribution, year was divided into four seasons: winter (January through March), spring (April through June), summer (July through September), and fall (October through December). Due to the inherent limitations of using presence-only information where observation effort and detectability are unknown, raw positions were simply mapped in their corresponding season, and no quantitative species distribution models were applied. In order to visualize shark distribution relative to typical SST conditions in the region, seasonal shark positions were overlaid on satellite-based 4 km Advanced Very High Resolution Radiometer (AVHRR) Pathfinder v.5.0 Seasonal Climatologies, averaged from 1985–2001 (National Oceanographic Data Center/University of Miami).

### Trends in abundance

Multiple historic and current data sources were examined for the presence of white sharks. Of those examined, we determined that only four data sources contained adequate information to estimate white shark trends in abundance for the NWA. Longline catch data were obtained from two sources: fishery-independent longline surveys conducted by the NMFS Northeast Fisheries Science Center (NEFSC) and its predecessor agencies between 1961 and 2009 [37]–[38] and the observer program of the directed shark bottom longline fishery from 1994–2010 [26], [39]. Data collected by the NMFS NEFSC at five recreational fishing tournaments from 1965 to 1996 (white sharks were listed as a NMFS prohibited species in 1997) were also used in this study. The tournaments were based out of New York (Bayshore Mako Tournament, Montauk Marine Basin Shark Tag Tournament, and Freeport Hudson Anglers, Inc. Shark Tournament) and New Jersey (Jersey Coast Shark Anglers Invitational Shark Tournament and South Jersey Shark Tournament). The final data source included sightings and capture records of white sharks in the NWA from 1800–2010 [14], [24], excluding records from the previous three time series, recent directed sightings effort, and accounting for historical directed effort leading up to and directly following the publication of the first comprehensive NWA white shark distribution paper [24]. Historical directed sightings effort was removed from the sightings time series during the late 1970s through the1980s based on the original datasheet notations and knowledge of the persons collecting the data during that time, resulting in an 80% reduction in these sightings records (Figure S1). Following initial analyses of the sightings data, additional sightings records in the vicinity of Monomoy Island, Massachusetts were removed in recent years for trend comparisons with respect to the increase in sightings near a growing population of gray seals (*Halichoerus grypus*) in that area [14].

Due to excess zero observations in the observer data, the fishery-independent longline surveys, and the tournament data, we used a mixture of a Bernoulli distribution (with a point mass of one at zero) for presence/absence data and a Poisson distribution for count data (including zeros) in a zero-inflated Poisson (ZIP) mixture model [40]–[41] to develop standardized indices of abundance. A number of parameters were considered as potential covariates affecting the presence/absence of white sharks and/or the white shark catch per set or tournament. For the NEFSC longline surveys, the variables available for consideration were year, season, depth, SST (<10°C, 10–14°C, 15–19°C, 20–24°C, >25°C), latitude, target (coastal shark, pelagic shark, pelagic inshore), bait type (teleost, elasmobranch, mixed), gear fishing on the bottom or up in the water column, leader type (wire, monofilament, mixed), hook number, and soak time. Variables available for the NEFSC tournament database were year, tournament, number of boats, number of days fished, and area (NY, NJ). For the observer program, the variables available for consideration were year, season, time of day, depth, area (Gulf of Mexico, southern Atlantic), hook type (small, medium, large, other), bait type (clupeid, elasmobranch, teleost, tuna, other), hook number, and research fishery participation (Amendment 2 to the 2006 Consolidated Highly Migratory Species Fishery Management Plan established a scientific research fishery in 2008 to gather information on *Carcharhinus plumbeus*). Stepwise forward model selection was used to determine which variables to retain in all final models based on the Akaike information criterion (AIC) and given a likelihood ratio test between the chosen model and the null model (intercept only) produced a test statistic value close to zero (≤0.01) [42]–[43. All models retained “year” in order to develop annual indices of abundance. Residual plots were used to determine the adequacy of model fits [43].

These standardized indices of abundance were then analyzed using a hierarchical framework to estimate a single time series of relative abundance [44]. This approach allows for the combination of multiple time series with differing lengths that do not all overlap in time [44]. The hierarchical approach developed by Conn [44] assumes that each index is measuring relative abundance and is subject to both process error and sampling error, the latter of which is presumably captured by the standardization process used to develop the indices of abundance. The indices (standardized to their means) and coefficients of variation were used in the hierarchical analysis to estimate individual index process error, assuming a lognormal error structure, and a hierarchical index of abundance [44]. The hierarchical analysis was conducted in a Bayesian framework using the same set of prior distributions as described by Conn [44] and used for other shark species for stock assessment purposes [45].

Annual white shark sightings were modeled using the approach developed by McPherson and Myers [16] to examine population trends from observational data. This method extracts the abundance trend in relative terms by fitting a series of generalized linear models to the difference in the count data between two points in time (difference between the most recent time point and any reference date) using a Poisson distribution and guards against sensitivity to unusually high or low counts by varying the reference period used to derive the count differences [16]. The estimated trend in relative abundance can then be viewed by plotting the magnitude of change in the number of reported sightings by year in log-space. Resulting values larger than 1 suggest an overall declining trend in abundance, values of 1 suggest a stable population, and values less than 1 suggest an overall increasing trend in abundance. This approach was used on the sightings data for multiple time frames. The sightings data were analyzed given any reference year from 1800 to 2008, 1950 to 2008, 1960 to 1986, and 1990 to 2008. Sensitivity analyses were conducted assuming changes in observation effort had either increased or decreased by 25% and 50% [16]. All analyses were conducted using the R programming environment [46].

## Results

We compiled a total of 649 verified white shark records from the NWA during the period 1800–2010. While the records date as far back as 1800, 94% occurred since 1950. Of these, 596 records had sufficient data (i.e., date and location) for seasonal distribution analysis and 433 were included in relative abundance time series runs (excluding directed effort, N = 200, and sightings with no associated year, N = 5).

**Figure 1:**
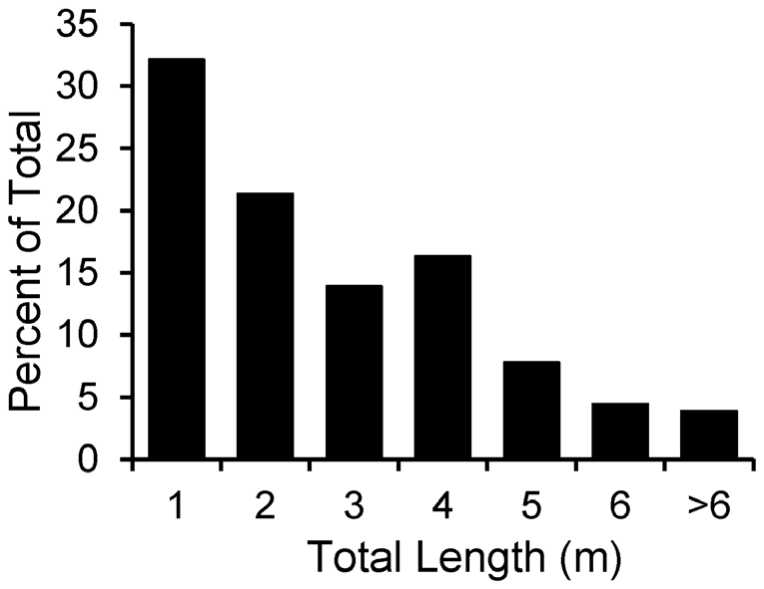
White shark lengths. Length frequency of white sharks from the western North Atlantic (N  =  538). These data include lengths from accurately measured specimens (N = 279), as well as estimated lengths, rounded down to the nearest m.

Sex of the shark was confirmed in 297 records and included 148 males and 149 females. Sharks that were accurately measured (N = 279) ranged in length from 1.22–5.63 m TL. An additional 259 records included estimated lengths, which we rounded down to the nearest m TL (1–9 m TL) (Figure 1). The records collectively included 124 YOY, 310 juveniles, and 104 mature sharks. While some white sharks were reported at estimated lengths exceeding 9 m, these estimations were considered unreliable. The largest shark considered accurately measured was a female specimen landed on Prince Edward Island, Canada in August 1983, which measured 5.26 m fork length (5.63 m TL).

### Gear interactions

**Figure 2:**
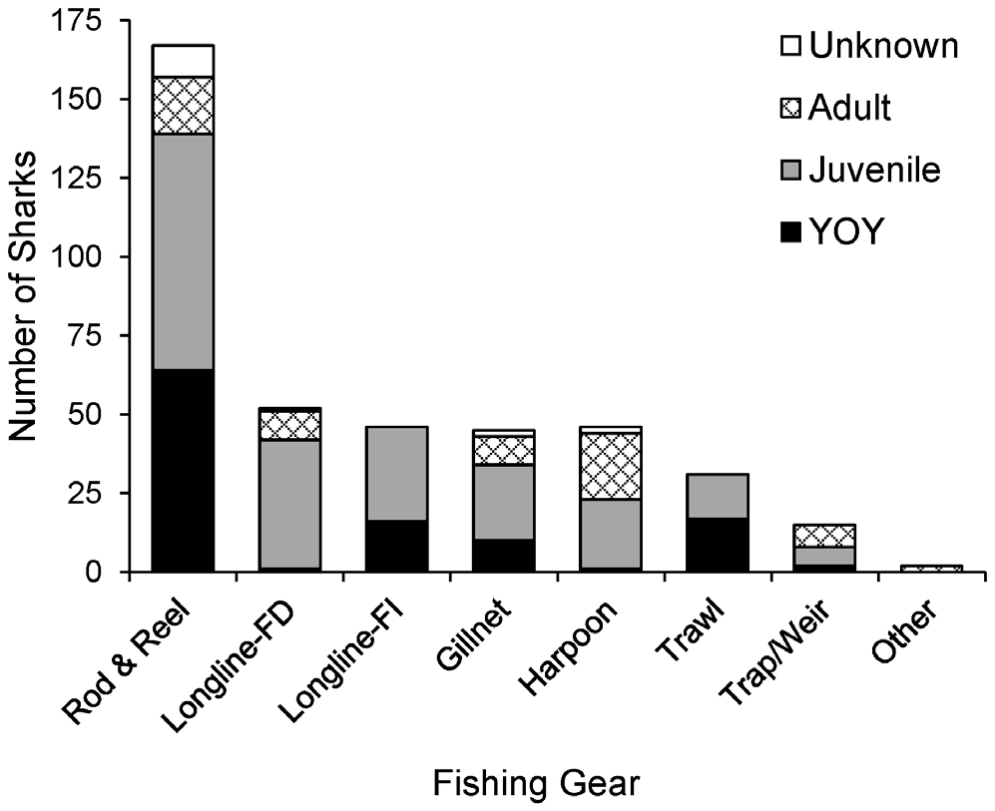
White shark gear interactions. Reported fishery-dependent and fishery-independent gear interactions with white sharks by life stage in the NWA, 1800–2009 (N = 390).

Confirmed gear interactions represented 66% (404) of the white shark records compiled, including both targeted and incidental catches. Forty-one percent of these records were derived from recreational rod and reel fishing (Figure 2). Amongst the remaining gear types, white sharks were most frequently captured by fishery-dependent (13%) and -independent (11%) longline gear (bottom and pelagic), harpoon (11%), and gillnet (11%, sink and drift), with fewer numbers caught in trawls (8%) and fish weirs/traps (4%, Figure 2). The practice of harpooning large white sharks, responsible for the majority (33%) of mature white shark captures, was more prevalent prior to 1980, and has been uncommon since 1997 when white sharks were prohibited from commercial and recreational harvest. Since 1985, fishery-dependent longline gear (40%) dominated reported white shark captures with rod and reel captures dropping to 35%. Within commercial fisheries (1985–2009), longline (60%) and gillnet (17%) have been the primary sources of incidental captures reported, and these gears predominantly catch immature sharks (Figure 2). Recreational rod and reel fishing accounted for 28% of the mature white sharks landed, with 72% of these captured between 1960 and 1990. Most of these landings occurred between Long Island, New York, and Massachusetts. However, juvenile white sharks (including YOY) were also frequently caught by rod and reel fishermen (Figure 2) targeting other large gamefish along the US coast.

### Seasonal distribution

**Figure 3:**
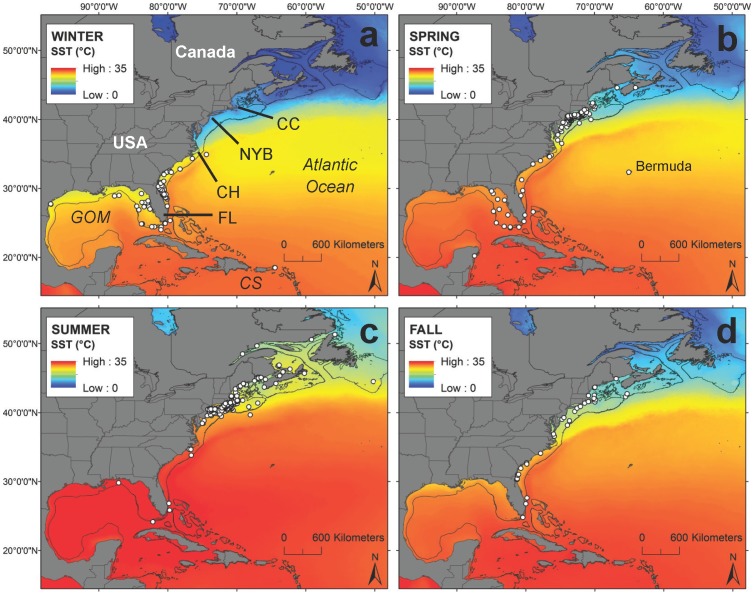
White shark seasonal distribution. Distribution of white shark presence records (white circles) in the NWA during (a) winter, (b) spring, (c) summer, and (d) fall. Positions are overlaid on seasonal average SST conditions (1985–2001). The 200 m bathymetric contour is displayed to delineate the edge of the continental shelf. CC  =  Cape Cod, NYB  =  New York Bight, CH  =  Cape Hatteras, FL  =  Florida, GOM  =  Gulf of Mexico, and CS  =  Caribbean Sea.

The range of white shark occurrence extended from the north coast of Newfoundland (51° N) to as far south as the British Virgin Islands (18° N), as far east as the Grand Banks (50° W) and Bermuda (65° W), to as far west as the coast of Texas in the Gulf of Mexico (97° W, Figure 3). While this overall distribution is quite broad, 90% of white sharks occurred along the US coast between 22° 00’ and 45° 30’ N (100% YOY, 86% juvenile, 89% mature). The center of distribution was in southern New England and the Mid-Atlantic Bight (between 35° 00’ and 42° 00’ N), where 66% of white sharks occurred (97% YOY, 54% juvenile, 70% mature).

**Figure 4:**
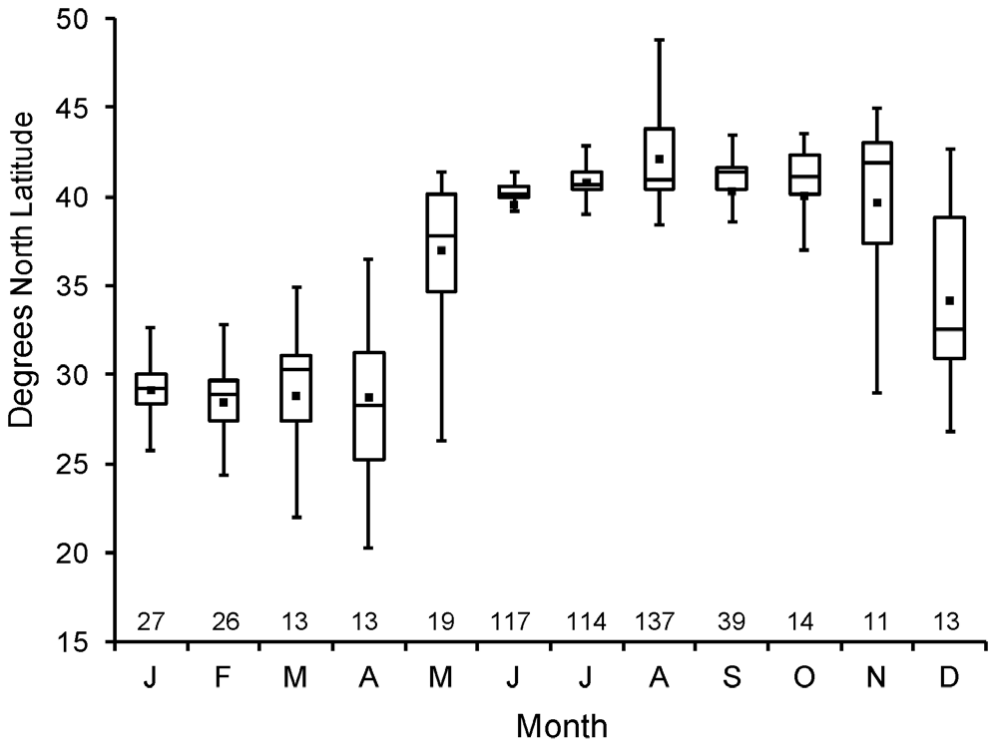
White shark monthly distribution. Box plots of latitudinal distribution of white shark presence by month in the NWA. The sample size in each month is given above the x-axis.

White sharks of all size/age classes were present in continental shelf waters throughout the year. However, there were considerable differences in distribution across seasons (Figure 3). During winter months, white sharks (2% YOY, 75% juvenile, 27% mature) were primarily distributed off the southeastern US and in the Gulf of Mexico (Figure 3a). Only one YOY white shark was captured during the winter months. This shark measured 1.64 m TL and was captured off North Carolina in January 1996. The median latitude of occurrence during winter months ranged from ∼28–31° N (Figure 4). No white sharks were reported north of Cape Hatteras (∼35° N) during winter (Figure 3a). Focal areas of winter occurrence were identified off the northeast coast of Florida (smaller juvenile through mature-sized individuals), off the Florida Keys (larger juvenile and mature sharks), and offshore of Tampa Bay (smaller juvenile through mature sharks) in the eastern Gulf of Mexico (Figure 3a).

During spring months, there was a clear expansion northward (Figure 3b, Figure 4). White sharks (28% YOY, 50% juvenile, 22% mature) occurred widely along the coast, mostly between the eastern Gulf of Mexico and the New York Bight (waters off the US Atlantic coast from Cape May Inlet in New Jersey to Montauk Point in Long Island, New York, Figure 3b). Median latitude of occurrence shifted dramatically across spring months, from 28° N in April to 40° N in June (Figure 4). The northernmost occurrences during this period typically occurred in late spring (May and June) (Figure 4) and the majority were large juvenile and mature sharks.

By summer, white sharks (23% YOY, 47% juvenile, 30% mature) appeared largely absent from southern coastal waters, occurring primarily in the Mid-Atlantic Bight, New England, and Canadian waters (Figure 3c). Only a few white sharks (mature) have been reported from south of Cape Hatteras during summer (Figure 3c). Most records were centered from the New York Bight eastward and north to Cape Cod. White sharks, predominately large juvenile and mature individuals, appear to reach the most northern portions of their NWA range (Newfoundland, Gulf of St. Lawrence) during August (Figure 4), but the median latitude of occurrence for all life stages remains around 40–41° N throughout the summer (Figure 4).

YOY sharks were most frequently encountered during summer between the central coast of New Jersey and Massachusetts Bay. However, most YOY shark observations (64%) were concentrated in the New York Bight between Great Bay, New Jersey, and Shinnecock Inlet, Long Island, New York. Neonate-sized white sharks (N = 46) were documented in this area between June and October (85% in June-August). Mature-sized female white sharks were also documented from this region during summer months, but no gravid or post-partum females were examined.

White sharks (15% YOY, 64% juvenile, 21% mature) remained in northern latitudes into the fall (Figure 3d), but appeared to begin a southward transition in November and December (Figure 4). Similar to spring months, white shark occurrence was broadly distributed along the coast between New England and the east coast of Florida (Figure 3d). The largest shift in median latitude occurred between November (42° N) and December (34° N, Figure 4).

### Habitat Use

**Figure 5:**
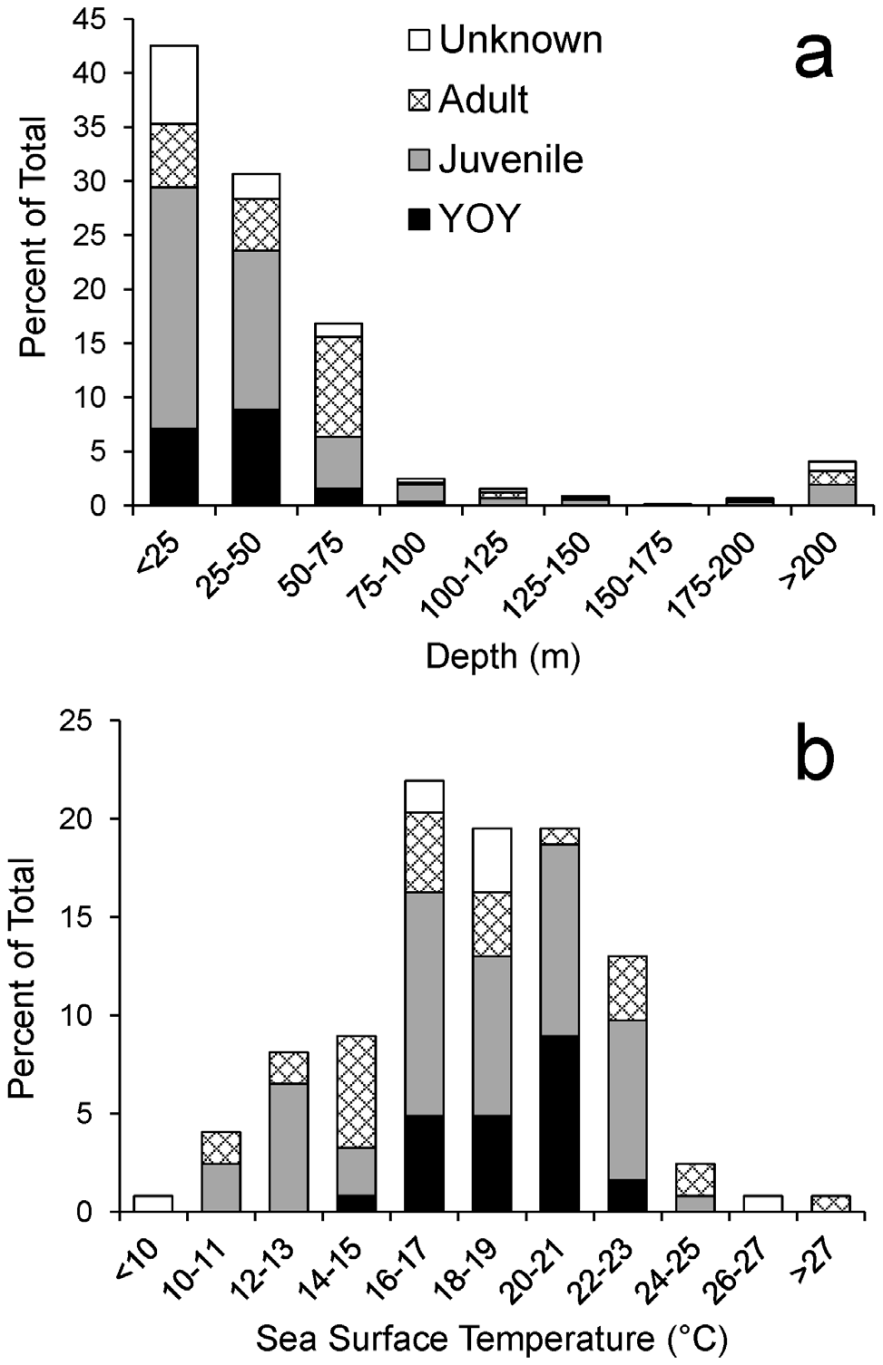
White shark habitat use. Distribution of (a) bottom depths (N = 564) and (b) SST (N = 124) associated with NWA white shark captures/sightings.

While environmental observations were limited throughout this data set, some patterns of habitat use were identified. Depth distribution data (N = 564) indicated that white sharks were predominantly encountered over continental shelf waters (200 m, Figures 3 and 5a). Over 92% of observations occurred in waters 100 m deep, and the median reported depth at occurrence was 30 m (mean ±1 SD  = 69±235 m). Only 23 observations occurred in deeper waters off the continental shelf, however, many of these were still relatively close to shore (e.g., off the Florida Keys, Figure 3). For YOY (N = 102), juvenile (N = 265), and mature (N = 125) sharks, the median depth at occurrence was 32 m (mean ±1 SD  = 32±19 m), 26 m (mean ±1 SD  = 45±74 m), and 50 m (mean ±1 SD  = 89±190 m), respectively; indicating a potential increase in the use of deeper waters by white sharks with increased size/age.

White sharks were captured in SSTs (N  = 124) of 9–28°C (mean ±1 SD  = 18.3±3.5°C). For YOY (N = 26), juvenile (N = 68), and mature (N = 21) sharks, the median reported SST at occurrence was 19.5°C (mean ±1 SD  = 19.0±1.9°C), 18°C (mean ±1 SD  = 18.1±3.5°C), and 16°C (mean ±1 SD  = 17.7±4.6°C), respectively. Over 80% of observations with temperature information were between 14 and 23°C (Figure 5b). Additionally, analysis of the NEFSC longline survey database suggested a preference for a similar SST range (see Trends in Abundance section).

### Trends in Abundance

The best fit model for the NEFSC longline surveys indicated that both the presence/absence and number of sharks per set were primarily influenced by soak time. There was a higher likelihood of catch with longer soak times, but within the positive catch sets, the longest soak times produced fewer white sharks, possibly due to bite offs (observed severed leaders) and/or predation. The presence/absence of white sharks in the NEFSC longline surveys was also influenced by SST with a higher likelihood of catch in the 15–19°C and 20–24°C temperature categories. Depth also influenced catch per set with higher catch rates in shallower depths. The presence/absence of a white shark at sampled tournaments was influenced by tournament location, with a higher likelihood of catching a white shark during one of the tournaments based out of New Jersey during the reported sampling time frame. For the observer program, the presence/absence of white sharks was primarily influenced by area fished and effort (number of hooks); catch per set was also influenced by area fished as well as season (highest catches off the Atlantic coast of Florida during the winter).

**Figure 6:**
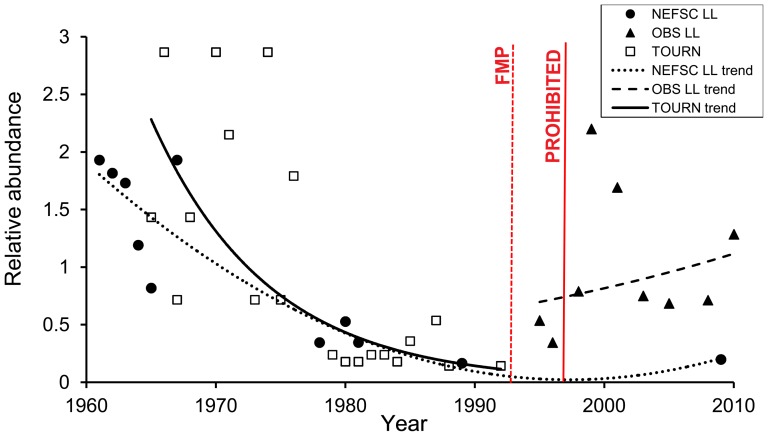
White shark relative abundance. White shark indices of abundance (index/mean) standardized using a zero-inflated Poisson model plotted by year for three time series: NEFSC LL  =  Northeast Fisheries Science Center fishery-independent longline surveys, TOURN  =  NEFSC tournament database, and OBS LL  =  observer program of the directed shark longline fishery. Trend lines are best fit regression models of the standardized data (second order polynomial for NEFSC LL and exponential for TOURN and OBS), using R^2^ values and considering the biology of the white shark. The dashed red line indicates the year of the first fishery management plan (FMP) for Atlantic sharks in 1993 [77] and the solid red line indicates the year that white sharks were listed as a NMFS prohibited species in 1997 [21].

Both standardized indices of relative abundance for the NEFSC longline surveys and the tournament data show decreasing estimates over time until the end of tournament time series, when white sharks were prohibited. Then the NEFSC longline index appears to increase based on best fit regression models of the data (Figure 6). The second order polynomial trend line estimated for this time series fits with our knowledge of the survey data in that the ZIP model could not provide estimates for several zero catch years during the mid to late 1990s and into the early 2000s. The observer index, which started after the implementation of the first shark fishery management plan in 1993, has an overall increasing trend in relative abundance throughout the time series, despite the large peak in the early 2000s, which the standardization process could not account for (Figure 6).

**Figure 7:**
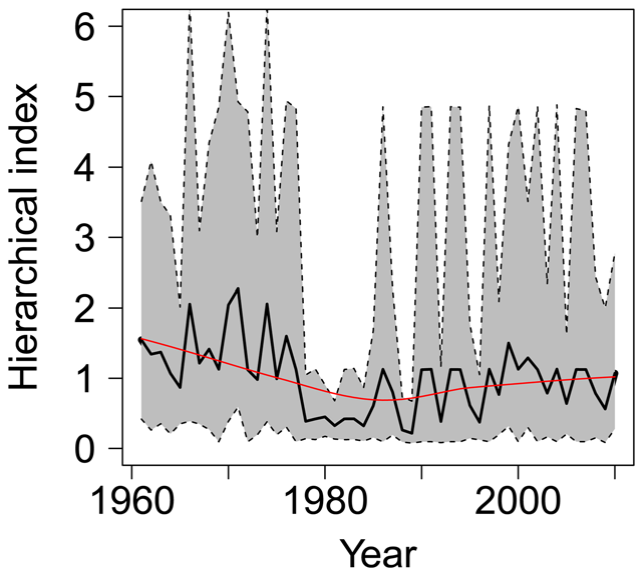
White shark relative abundance trend. Time series of white shark relative abundance in the NWA as estimated from hierarchical analysis. The continuous black line gives the posterior mean, and the shaded area represents a 95% credible interval about the time series. The red line is the estimated trend based on locally weighted polynomial regression using the LOWESS smoother.

The hierarchical trend combining all three indices, although slightly masked by the large credible intervals for the index, shows historically higher abundances during the 1960s and into the mid-1970s with a declining trend into the late 1980s and then begins a gradual increasing trend through the remainder of the time series (Figure 7). During the mid-1970s and throughout the 1980s, white shark relative abundance had declined between 27 and 86%, with a median value of 73%. The most recent year in the time series (2010) shows only a 31% decline in white shark abundance from its historical abundance estimate in 1961. Estimates of process error show the three indices performed reasonably well for white shark abundance and values were similar across indices (indices process standard deviation estimates ranged from 0.405–0.457, Figure S2).

**Figure 8:**
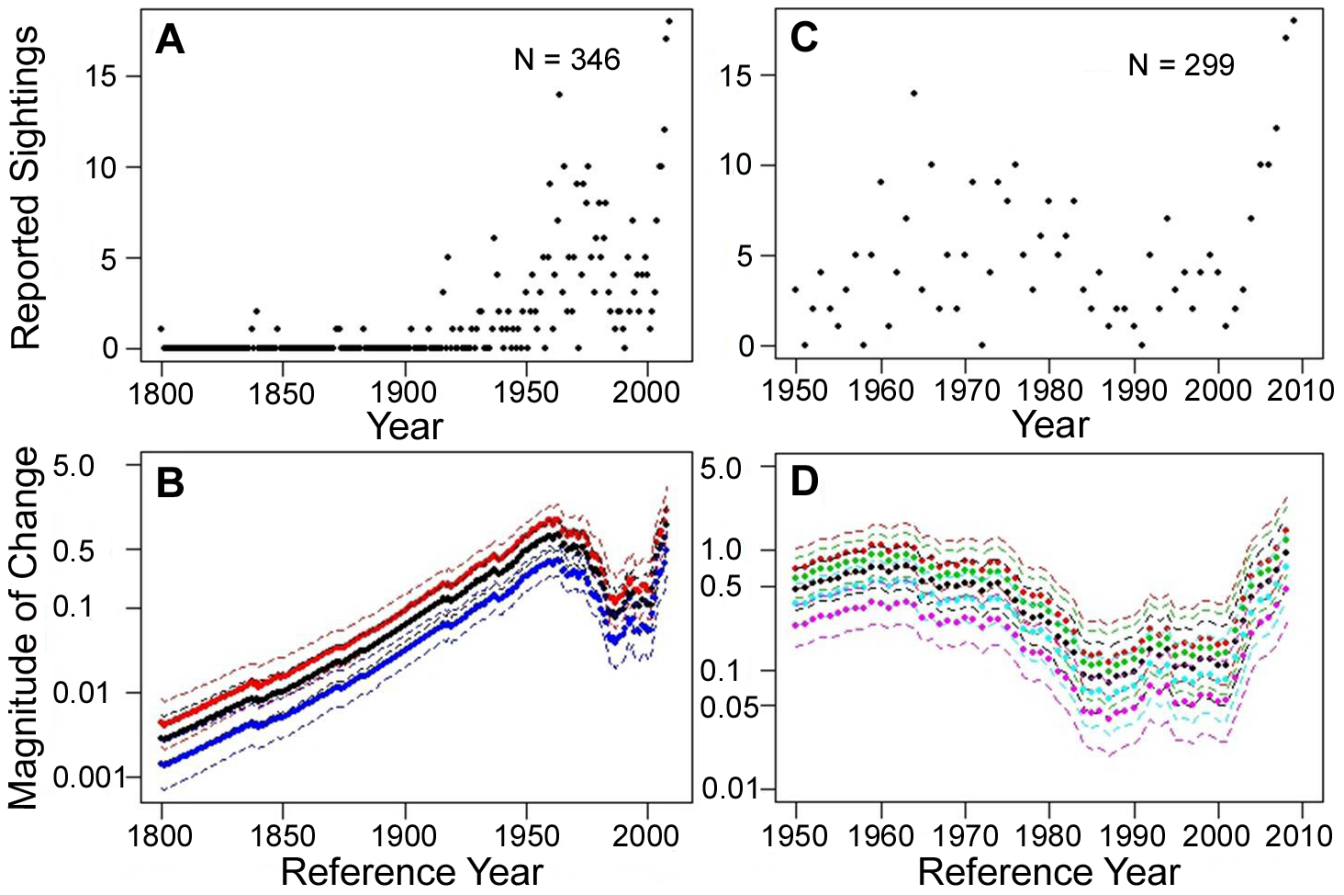
Time series of white shark sightings. (a) Number of annual white shark sightings reported in the NWA from 1800 to 2009. (b) Estimates of relative change in abundance (filled circles) with 95% credible intervals (dashed lines) for any reference year between 1800 and 2008 assuming no change (black plot), a 50% increase (red plot), and a 50% decrease in observation effort. (c) Number of annual white shark sightings reported in the NWA from 1950 to 2009. (d) Estimates of relative change in abundance (filled circles) with 95% credible intervals (dashed lines) for any reference year between 1950 and 2009 assuming no change in observation effort (black plot), a 25% and 50% increase in observation effort (green and red plots, respectively), and a 25% and 50% decrease in observation effort (blue and purple plots, respectively).

**Figure 9:**
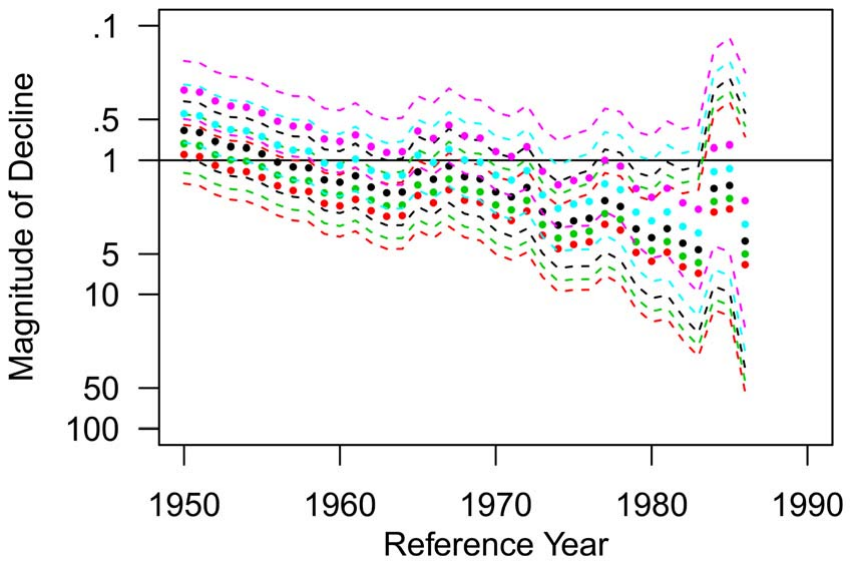
White shark relative decline in abundance. Estimates of relative decline in abundance (filled circles) with 95% credible intervals (dashed lines) for any reference year between 1960 and 1986 assuming no change in observation effort (black plot), a 25% and 50% increase in observation effort (green and red plots, respectively), and a 25% and 50% decrease in observation effort (blue and purple plots, respectively). Note that the scale for the y-axis has been reversed when compared to Figure 8 to visualize the declining trend in abundance during this time period.

**Figure 10:**
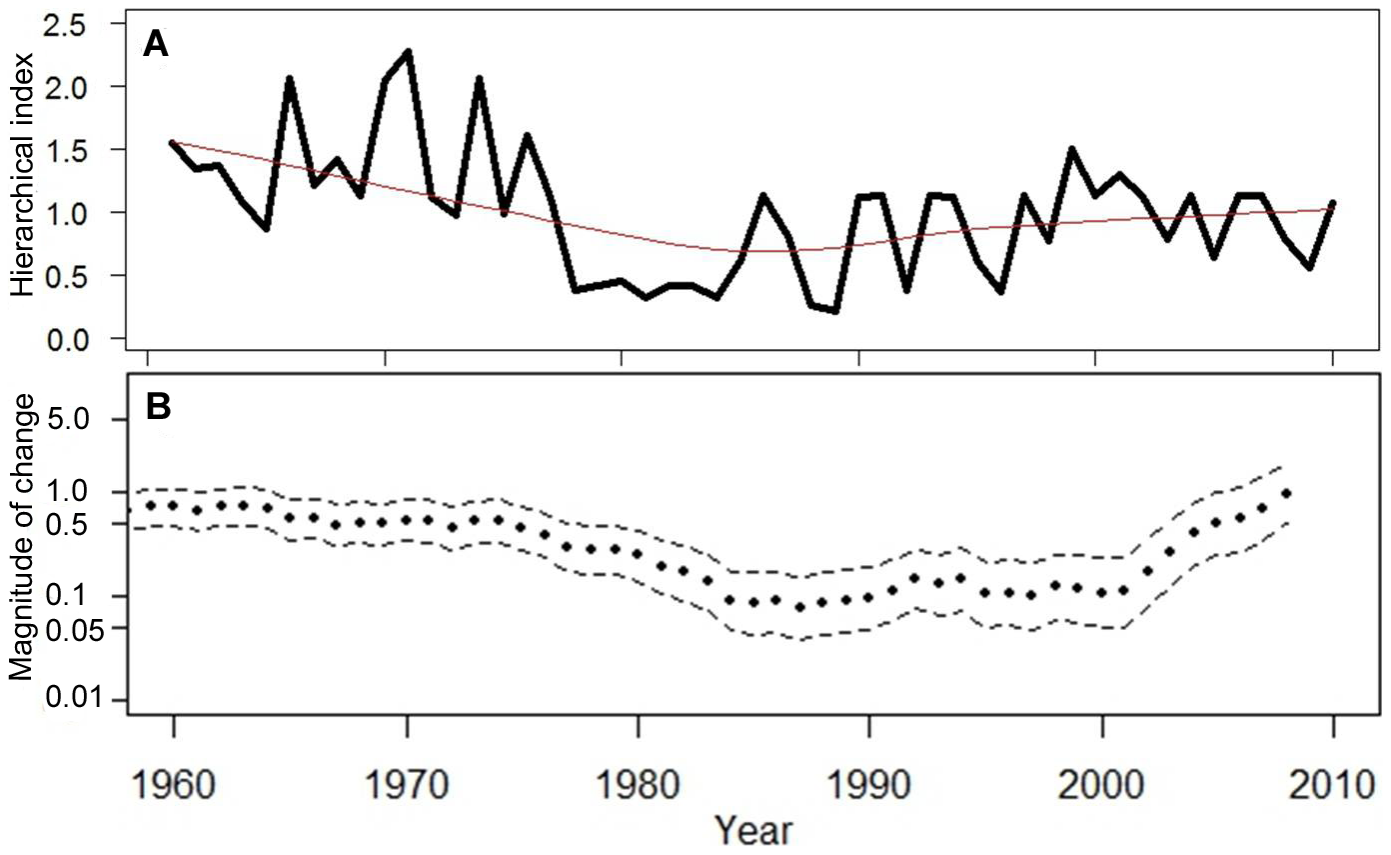
Trend comparison of white shark relative abundance. (a) Estimated trend from the hierarchical analysis, and (b) estimated trend from the sightings analysis.

Excluding the time series analyzed separately and directed effort, a total of 346 white sharks were sighted between 1800 and 2009 (Figure 8a), with over 86% (299) of the sightings occurring between 1950 and 2009 (Figure 8c). Under the assumption of no change in observational effort, the sightings model estimated that there was an overall increasing trend (all estimated values less than 1) in the NWA white shark population since the 1800s, most notably during the beginning of the time series through the 1950s and during more recent years (Figure 8b). A closer look at the relative abundance trend starting in the 1950s, reveals that even though the change in magnitude from any reference year between 1950 and 2008 to the terminal year in 2009 results in an increase in relative abundance (magnitude of change 1), there still appears to be a declining trend during the 1970s into the mid 1980s (Figure 8d). Sensitivity analyses estimating 25 and 50% increases and decreases in observation effort clearly increases the uncertainty surrounding the estimates of change in abundance, but the overall trend remains the same. Analysis of the sightings data with a terminal year of 1987 reveals an estimated 2–4-fold (median estimate  = 2.71, 63% decline) decrease in the population since any reference year between 1970 and 1986 (Figure 9). If we reduce the observational effort by 25% and 50%, it reduces the estimated decline during the 1970s into the mid 1980s to 51% and 26%, respectively (median estimates  = 2.02 and 1.36, respectively, Figure 10). A 98% reduction in observational effort is needed to avoid a decline in abundance during that time frame (model estimates and confidence bounds consistently drop below 1). During the 1990s, the relative abundance trend appears to stabilize and then begins an increasing trend during the 2000s until the end of the time series (Figures 8b, 8d). This overall increasing trend in relative abundance during the end of the time series is retained when assuming 25 and 50% increases and decreases in observation effort (Figures 8b, 8d).

**Figure 11:**
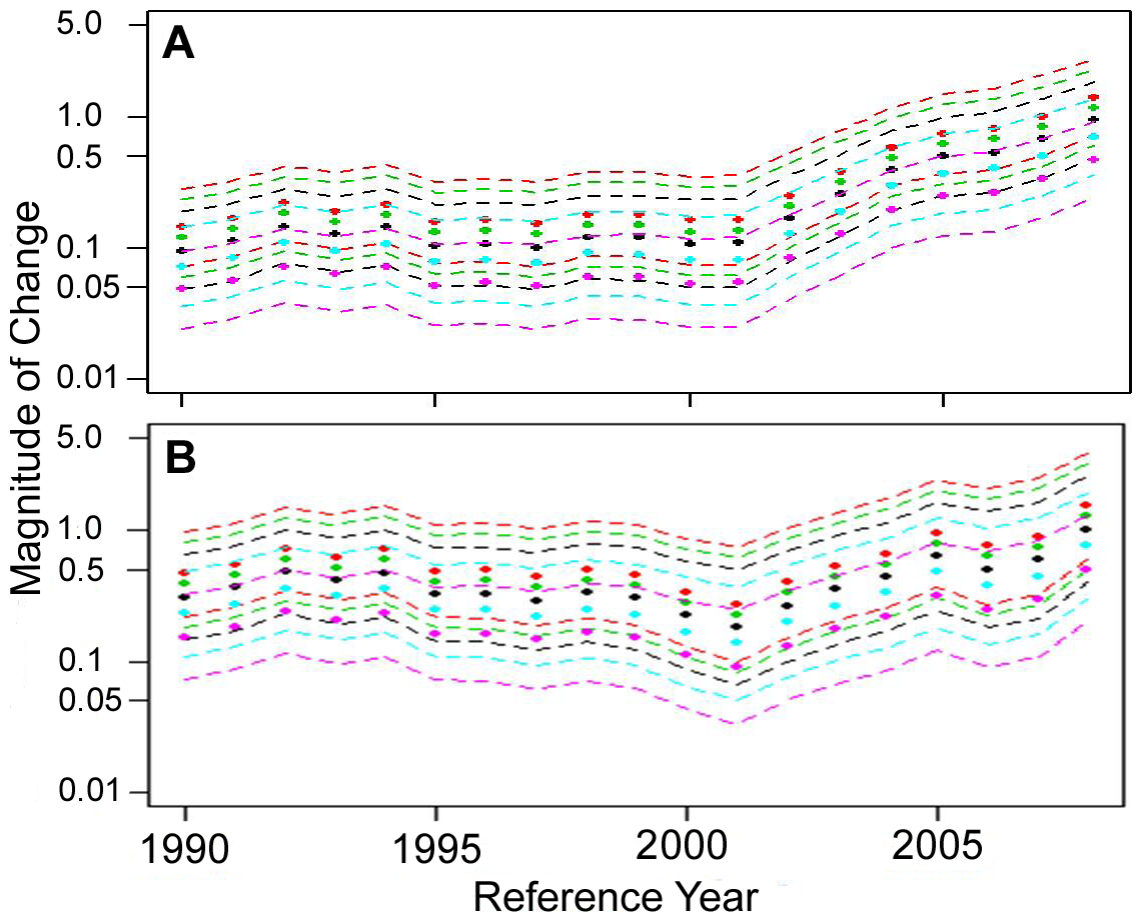
Recent trends in white shark relative abundance. Estimates of relative change in abundance (filled circles) with 95% credible intervals (dashed lines) for any reference year between 1990 and 2008 assuming no change in observation effort (black plot), a 25% and 50% increase in observation effort (green and red plots, respectively), and a 25% and 50% decrease in observation effort (blue and purple plots, respectively) for the original sightings time series from 1990 to 2009 (a) and the time series with sightings that occurred near Monomoy Island during that time frame removed (b).

A comparison of abundance trends between the hierarchical and sightings methods reveals a strikingly similar pattern except at the end of the time series, where the sightings time series has a much steeper increase in abundance (Figure 10). Removal of white shark sightings from the sightings data during the 1990s and 2000s around a growing gray seal colony on Monomoy Island still provides an increasing trend, but an overall smaller magnitude of change and results in a more gradual slope that is more in line with the trend estimated for the hierarchical index (Figures 10, 11).

## Discussion

This study represents the most comprehensive synthesis of data on NWA white sharks to date, and significantly updates previous reviews [24]–[25]. In general, the white shark remains an uncommon and sparsely distributed predator in the NWA. However, by combining over two centuries worth of observations the results have provided new insights into population and distribution trends along the east coast of North America.

### Seasonal Distribution and Habitat

The use of presence-only data for describing species distributions has inherent limitations (e.g., [47]). Results may be biased by spatial and temporal variability in observation effort, detectability, and catchability [16], [47]. However, presence records from captures and sightings are often the best source of baseline information on comparatively uncommon marine species like the white shark [10], [24], [48]–[49]. Since the majority of our records were derived from fisheries interactions, patterns in fishing effort and gear over space and time should partially account for the patterns we have described. One important bias is that the occurrence of adult white sharks in our dataset is likely underestimated due to the fact that these large individuals can more easily escape entanglements/hooking in fishing gear.

Since most fishing effort and boating activity in the NWA occurs over continental shelf waters, encounter rates with white sharks may be biased toward the coasts. Therefore, white shark occurrence in offshore waters may be underrepresented in this analysis. The only fishery likely to encounter white sharks in offshore waters is the pelagic longline fishery, which targets tunas and swordfish, but regularly incidentally captures pelagic shark species including silky (*Carcharhinus falciformis*), dusky (*C. obscurus*), oceanic whitetip (*C. longimanus*), and blue (*Prionace glauca*) sharks [15], [50]. However, the occurrence of white sharks in this offshore fishery appears to be extremely low [24], [50]–[51]. We agree with the assertions of Burgess et al. [17] that the 6,087 white sharks reported in pelagic longline fishery logbooks according to Baum et al. [15] were probably not in fact *Carcharodon carcharias*, and these records should not be used to infer distribution or abundance patterns for this species. Given the occasional reports of white sharks from offshore waters beyond the continental shelf, including their documented occurrence in Bermuda waters [33] and recent satellite tracking data (GBS, unpublished data), further observations, stable isotope analyses, and/or advanced technology tagging studies are needed to provide a greater understanding of their use of offshore habitats in this region.

In the absence of seasonal shifts in shark distribution, fisheries would be expected to have fairly equal probability of encountering white sharks across the year throughout their range. However, this was not the case for several fisheries, as encounters were unevenly distributed across seasons. For example, despite observer coverage for the majority of the year in the shark bottom longline fishery [26], [39], no white sharks were encountered during summer months off the southeast US. Likewise, catch and observer records in commercial trawl and gillnet fisheries off New England and Canada primarily documented white sharks during summer months, despite year-round fishing activity and observer coverage (NMFS Northeast Fisheries Observer Program, unpublished data). These trends appear to support the seasonal north-south distribution shift of the NWA white shark population, despite the limitations of using presence-only information. This north-in-summer, south-in-winter distributional pattern is typical of numerous temperate, coastal, migratory fishes in the northern hemisphere (e.g., [52]–[53]) and white shark migrations from temperate to subtropical waters have also been documented off the west coast of the United States and Mexico [54]–[55] and off the Pacific coasts of Australia and New Zealand [56]–[57].

Consistent with previous studies on white sharks (e.g., [18], [24], [31]), temperature appears to exert a significant influence on distribution, and is likely a key migratory cue in the region. The seasonal movement of the white shark population up and down the Atlantic coast of North America, an average shift of approximately 12° of latitude (28–40° N, Figure 4), allows white sharks to remain within an apparently preferred SST range of ∼14–23°C. Given their comparatively large body mass and endothermic capabilities [58], this relatively narrow temperature range does not define the white sharks thermal tolerance which extends from at least 3–28°C [55], [59]–[60], but it does appear to largely define the bounds of their seasonal latitudinal range in this region. Therefore, while temperature may drive seasonal distribution shifts, the selection of specific summer and winter habitats is likely based upon environmental characteristics secondary to temperature (e.g., prey availability).

The relatively broad summer focal area for white sharks between the coasts of New Jersey and Massachusetts likely include important foraging areas across life stages. YOY and juvenile white sharks, which were more prevalent in the New York Bight region during summer, would have access to a wide variety of demersal and pelagic teleosts and elasmobranchs for prey [24]. The waters less than 50 m deep on the broad continental shelf in the New York Bight area may represent primary nursery habitat for YOY white sharks [24]. The seasonal peak in the presence of neonate-sized sharks suggests that parturition may occur near this area between May and August. White shark nursery habitat has also been identified in other regions along continents where larger expanses of shelf habitat exist [56], [61].

Large white sharks (3.0 m) tend to preferentially feed upon marine mammals including pinnipeds, small cetaceans, and large whale carcasses [10], [18], [62]–[63]. Since pinniped populations in the NWA have been severely depressed throughout most of the last century [64], confirmed predations on seals (*Phoca vitulina*, *Halichoerus grypus*) have been rare until very recently [14], [32], [65]. Whale carcasses are thought to be one of the most important sources of food for large white sharks in this region [66]. White sharks have been observed scavenging dead whales off New England and Long Island, New York on numerous occasions [24,63, 66–67, NMFS unpublished data, JKC personal observation), but they also supplement their diet with odontocete whales such as the harbor porpoise (*Phocoena phocoena*) [68]–[69] and fishes including tunas (Thunnus spp.), sea robins (*Prionotus* spp.), menhaden (*Brevoortia tyrannus*), hakes (*Urophycis* spp.), skates (Rajidae), bluefish (*Pomatomus saltatrix*), smooth dogfish (*Mustelus canis*), and other shark species ([24], NMFS unpublished data).

Due to the dynamic and broad distribution of prey (i.e., teleosts, marine mammals) in this region, white sharks must forage over a broad area, rather than at discrete aggregation sites like those off California, Australia, or South Africa (e.g., [70]–[72]). However, the recovery of NWA gray seal populations over the last decade [64] and their increasing concentrations at specific sites along Cape Cod, Massachusetts, appears to be producing new localized summer feeding aggregations for white sharks [14].

Although the summer distribution of white sharks in the NWA has been described in previous studies [24]–[25], there has been very limited information on the focal areas for white shark occurrence during winter months. White sharks have long been thought to be rare and occasional visitors to coastal waters off the southeast US, Gulf of Mexico, and the northern Caribbean Sea [24], [28]–[31]. However, the current results indicate that white sharks visit these subtropical waters on a regular basis during the winter. The most notable areas of repeated occurrence during winter months are the Atlantic shelf waters between southern Georgia and Cape Canaveral, Florida and Gulf of Mexico shelf waters west of Tampa Bay, Florida for small juvenile through mature sized individuals, and Atlantic coastal waters along the Florida Keys for larger juvenile and mature white sharks.

The reasons why white sharks are drawn to particular subtropical areas during winter months are unclear, but they likely include important foraging grounds. Analysis of white shark stomach contents from this region are extremely limited, however, documented prey items include dolphins (Delphinidae), sharks (Carcharhinidae), red drum (*Sciaenops ocellatus*), sea turtles, and squid ([31], Authors unpublished stomach contents data). Historically, white sharks that occurred along the Florida Keys and northern Caribbean islands may have also preyed upon the now extinct Caribbean monk seal (*Monachus tropicalis*) [73]. Juvenile and adult white sharks have also been observed scavenging upon the carcasses of North Atlantic right whales (*Eubalaena glacialis*) in the waters off Georgia and northern Florida on several occasions [74]. This area is designated as critical habitat for the right whale, and includes their primary (December-March) calving grounds [75]. White sharks are not known to actively prey upon healthy adult mysticete whales [63], [76], but it is possible that they are drawn to this area during the right whale calving season in order to attempt to prey upon calves [74], or scavenge upon occasional carcasses of adults or calves and/or whale placentas. Seasonal movement of white sharks to subtropical calving grounds of humpback whales (*Megaptera novaengliae*) has been documented in the North and South Pacific Oceans (e.g., [49], [54], [57]). Despite the unpredictable availability of large whale carcasses, white sharks may regularly migrate to whale aggregation areas for foraging/scavenging. The particularly high caloric value of whale blubber tissue [66] makes it an optimal food choice to help meet the high energetic demands of the endothermic white shark [58], [63].

In summary, given the available information on white shark distribution, feeding habits, and habitat use, it appears that the annual north-south distribution shift of the white shark population is driven by a combination of environmental preferences and prey availability. White sharks move into summer feeding areas off the northeast US when SST rises above approximately 14°C. They feed on a wide variety of prey over a broad area, but large white sharks have been increasingly associated with emerging gray seal colonies off Massachusetts in recent years [14]. As temperatures decline during the fall, the shark population shifts southward, eventually reaching putative foraging grounds off Georgia and Florida. White sharks have been documented to occur on continental shelf waters throughout the year, and may migrate along the Atlantic coast rather than regularly moving into offshore pelagic waters, as they do in the eastern North Pacific (e.g., [54]–[55]). The sparse observations in Mid-Atlantic waters between Maryland and South Carolina for all life stages suggest this stretch of coast may be a migratory corridor, connecting northern and southern feeding areas. However, preliminary satellite tracking data from this region suggest that some individuals may also spend considerable amounts of time beyond the continental shelf (GBS, unpublished data). More observations, tagging, and telemetry studies are necessary to shed more light on these patterns.

### Abundance Trends and the Status of NWA White Sharks

The results of our relative abundance analyses offer a more optimistic outlook for NWA white sharks than previous reports [15], [16]. Consistent with previous analyses, significant declines (63–73%) through the 1970s and 1980s were identified, but previously undocumented positive trends were present in available time series since the early 1990s. The hierarchical method, allowing the combination of multiple time series that did not all overlap in time, had the largest amount of uncertainty associated with its estimated trend of relative abundance. During simulation testing of the hierarchical method, Conn [44] reported that the credible intervals for the hierarchical index were frequently wider than nominal for all simulation scenarios, suggesting that the estimation procedure was overly conservative. Although there is uncertainty in all trends used in this study, the concordance of multiple data sources in the timing of population changes lends credence to the observed patterns. The population declines of the 1970s and 1980s and the increases during the 1990s are also parsimonious with our understanding of the expansion and eventual regulation of shark fisheries during this period [21], [27], [77].

Though no real trend can be inferred, an additional source of historic and contemporary relative abundance comes from the shark bottom longline fishery off Florida [24], [26], [51]. From 1935–1950, prior to widespread commercial shark fishing and purported population declines, white sharks represented approximately 1 out of every 3,704 sharks captured in this fishery [24], [51]. Despite some likely changes to gear and effort over time, Morgan et al. [26] reported that white sharks represented approximately 1 out of every 3,443 sharks captured in the same fishery between 1994 and 2003, a remarkably small difference between observations separated by over 40 years. Though these are just two points in time, the similarity in relative occurrence may indicate that white shark abundance in this region is currently comparable to what it was in the 1930s and 1940s. Had the stock collapsed and remained at decimated levels, the relative occurrence ratio in Morgan et al. [26] would likely have been significantly lower than that reported by Springer [51].

There is evidence suggestive of recent increases in white shark abundance in other regions, similar to what is documented here for the NWA. Catch per unit effort from protective beach nets show an apparent increasing trend in relative abundance for white sharks during the 2000s in South Africa [78] and during the mid 1990s through the 2000s in New Zealand [79]. Catches of white sharks from southern California fisheries have also increased in recent years despite significant reductions in fishing effort [12]. Similar to the US Atlantic, all of these regions have legally protected white sharks from harvest since the 1990s. Though data remain comparatively sparse for white sharks, and significant uncertainty remains in all abundance trend estimates ([12], [16], [78]–[79], this study), there is growing evidence that legal protections for white sharks in the NWA and elsewhere around the world have been effective. Population declines appear to have been halted and populations may now be stabilized or growing in several regions. However, given the white sharks inherent sensitivity to exploitation and low productivity [4], [9], fishery bycatch mortality remains a concern to the long-term sustainability of their populations.

Despite some recent progress in our understanding of the biology of white sharks in the NWA ([9], [14], [74], [80], this study), there are still considerable knowledge gaps in this region compared to other areas [23]. Significant questions remain on life history, population structure and size, behavior, habitat preferences, feeding habits, movements, and migration. Other than the possible presence of a summer nursery area in the New York Bight, virtually nothing is known about the location and timing of mating or parturition. It is not known if the timing and extent of white shark migrations in the NWA are similar to those described in recent satellite tracking studies in the Pacific and Indian Oceans [54]–[55], [59], [81]. Further research will help fill in many of these information gaps, and continued compilation of opportunistic sightings, fishery captures, and examination of occasional specimens will, over time, help to further expand our knowledge and improve conservation strategies.

## Supporting Information

Figure S1 **Time series of white shark sightings.** (a) Number of annual white shark sightings reported in the NWA from 1800 to 2009, excluding the time series used in the hierarchical analysis and recent directed effort. The vertical red line indicates the year the first comprehensive NWA white shark distribution paper was published [24]. (b) Number of annual white shark sightings used to model trends in abundance, contains an 80% reduction in records leading up to and directly following the Casey and Pratt [24] publication (red line) to account for directed effort during that time.
(TIF)

Figure S2 **Process errors for white shark relative abundance indices.** Posterior means and 95% credible intervals for the standard deviation (SD) of process error for the three indices used in the hierarchical analysis. NEFSC LL  =  Northeast Fisheries Science Center fishery-independent longline surveys, TOURN  =  NEFSC tournament database, and OBS LL  =  observer program of the directed shark longline fishery.
(TIF)

## References

[1] Compagno LJV (2001) Sharks of the world: an annotated and illustrated catalogue of shark species known to date. Vol. 2. Bullhead, mackerel, and carpet sharks (Heterodontiformes, Lamniformes and Orectolobiformes). FAO Species Catalogue for Fishery Purposes, No. 1, Vol. 2. Rome, FAO. 269 pp.

[2] Cortés E (1999) Standardized diet compositions and trophic levels of sharks. ICES J Mar Sci 56: 707–717.

[3] Estrada JA, Rice AN, Natanson LJ, Skomal GB (2006) Use of isotopic analysis of vertebrae in reconstructing ontogenetic feeding ecology in white sharks. Ecol 87(4): 829–834. 10.1890/0012-9658(2006)87[829:uoiaov]2.0.co;2 16676526

[4] Smith SE, Au DW, Show C (1998) Intrinsic rebound potentials of 26 species of Pacific sharks. Mar Freshw Res 49: 663–678.

[5] Cortés E (2002) Incorporating uncertainty into demographic modeling: Application to shark populations and their conservation. Conserv Biol 16(4): 1048–1062.

[6] Chapple TK, Jorgensen SJ, Anderson SD, Kanive PE, Klimley AP (2011) A first estimate of white shark, *Carcharodon carcharias*, abundance off Central California. Biol Lett doi:10.1098/rsbl.2011.0124. PMC3130246 21389017

[7] Francis MP (1996) Observations on a pregnant white shark with a review of reproductive biology. In: Klimley AP, Ainley DG, editors. Great white sharks: The biology of *Carcharodon carcharias*. San Diego: Academic Press. pp. 157–172.

[8] Wintner SP, Cliff G (1999) Age and growth determination of the white shark, *Carcharodon carcharias*, from the east coast of South Africa. Fish Bull 97(1): 153–169.

[9] Hamady LL, Natanson LJ, Skomal GB, Thorrold SR (2014) Vertebral bomb radiocarbon suggests extreme longevity in white sharks. PLoS ONE 9(1): e84006 10.1371/journal.pone.0084006 24416189 PMC3885533

[10] Klimley AP (1985) The areal distribution and autoecology of the white shark, *Carcharodon carcharias*, off the west coast of North America. S Cal Acad Sci, Mem 9: 15–40.

[11] Ellis R, McCosker JE (1991) Great white shark. New York: Harper Collins/Stanford University Press. 270 p.

[12] Lowe CG, Blasius ME, Jarvis ET, Mason TJ, Goodmanlowe GD (2012) Historic fishery interactions with white sharks in the Southern California Bight. In: Domeier ML, editor. Global perspectives on the biology and life history of the great white shark. Boca Raton: CRC Press. pp. 169–185.

[13] Santana-Morales O, Sosa-Nishizaka O, Escobedo-Olvera MA, Onate-Gonzalez EC, OSullivan JB (2012) Incidental catch and ecological observations of juvenile white sharks, *Carcharodon carcharias*, in western Baja California, Mexico: Conservation implications. In: Domeier ML, editor. Global perspectives on the biology and life history of the great white shark. Boca Raton: CRC Press. pp. 187–198.

[14] Skomal GB, Chisholm J, Correia SJ (2012) Implications of increasing pinniped populations on the diet and abundance of white sharks off the coast of Massachusetts. In: Domeier ML, editor. Global perspectives on the biology and life history of the great white shark. Boca Raton: CRC Press. pp. 405–417.

[15] Baum JK, Myers RA, Kehler DG, Worm B, Harley SJ (2003) Collapse and conservation of shark populations in the northwest Atlantic. Science 299: 389–392. 12532016 10.1126/science.1079777

[16] McPherson JM, Myers RA (2009) How to infer population trends in sparse data: examples with opportunistic sighting records for great white sharks. Divers Distrib 15: 880–890.

[17] Burgess GH, Beerkircher LR, Cailliet GM, Carlson JK, Cortes E (2005) Is the collapse of shark populations in the northwest Atlantic Ocean and Gulf of Mexico real? Fisheries 30 (10): 20–26.

[18] Cliff G, Dudley SFJ, Davis B (1989) Sharks caught in the protective gillnets off Natal, South Africa: 2. The great white shark *Carcharodon carcharias* (Linnaeus). S Afr J Mar Sci 8: 131–144.

[19] Reid DD, Krogh M (1992) Assessment of catches from protective shark meshing off New South Wales beaches between 1950 and 1990. Austr J Mar Freshw Res 43: 283–296.

[20] IUCN (2012) IUCN Red List of Threatened Species. Version 2013.1. URL http://www.iucnredlist.org.

[21] National Marine Fisheries Service (1997) Framework seasonal adjustment of management measures under the fishery management plan for sharks, final environmental assessment and regulatory impact review/final regulatory flexibility analysis.United States Department of Commerce, National Oceanic and Atmospheric Administration, National Marine Fisheries Service, Office of Sustainable Fisheries, Silver Spring, Maryland .

[22] Klimley AP, Ainley DG, editors (1996) Great white sharks: The biology of *Carcharodon carcharias*. San Diego: Academic Press. 517 p.

[23] Domeier ML, editor (2012) Global perspectives on the biology and life history of the great white shark. Boca Raton: CRC Press. 543 p.

[24] Casey JG, Pratt Jr HL (1985) Distribution of the white shark, *Carcharodon carcharias*, in the western North Atlantic. S Cal Acad Sci, Mem 9: 2–14.

[25] COSEWIC (2006) COSEWIC assessment and status report on the white shark *Carcharodon carcharias* (Atlantic and Pacific populations) in Canada. Committee on the Status of Endangered Wildlife in Canada. Ottawa, vii (www.sararegistry.gc.ca/status/status_e.cfm).

[26] Morgan A, Cooper PW, Curtis TH, Burgess GH (2009) Overview of the U.S. east coast bottom longline shark fishery, 1994–2003. Mar Fish Rev 71 (1): 23–38.

[27] Carlson JK, Hale LF, Morgan A, Burgess GH (2012) Relative abundance and size of coastal sharks derived from commercial shark longline catch and effort data. J Fish Biol 80(5): 1749–1764. 22497406 10.1111/j.1095-8649.2011.03193.x

[28] Springer S (1939) The great white shark, *Carcharodon carcharias* (Linnaeus), in Florida waters. Copeia 1939: 114–115.

[29] Clark E, von Schmidt K (1965) Sharks of the central Gulf coast of Florida. Bull Mar Sci 15: 13–83.

[30] Rivas LR, McClellan DB (1982) Shark investigations by the National Marine Fisheries Service, Miami Laboratory. Fla Sci 45: 40–45.

[31] Adams DH, Mitchell ME, Parsons GR (1994) Seasonal occurrence of the white shark, *Carcharodon carcharias*, in waters off the Florida west coast, with notes on its life history. Mar Fish Rev 56 (4): 24–28.

[32] Mollomo P (1998) The white shark in Maine and Canadian waters. Northeast Nat 5 (3): 207–214.

[33] Smith-Vaniz WF, Collette BB, Luckhurst BE (1999) Fishes of Bermuda: History, zoogeography, annotated checklist, and identification keys. American Society of Ichthyologists and Herpetologists Special Publication No. 4.

[34] Mollet HF, Cailliet GM, Klimley AP, Ebert DA, Testi AD (1996) A review of length validation methods and protocols to measure large white sharks. In: Klimley AP, Ainley DG, editors. Great white sharks: The biology of *Carcharodon carcharias*. San Diego: Academic Press. pp. 91–108.

[35] Kohler NE, Casey JG, Turner PA (1996) Length-length and length-weight relationships for 13 shark species from the western North Atlantic. NOAA Tech Memo NMFS-NE-110.

[36] Pratt Jr HL (1996) Reproduction in the male white shark. In: Klimley AP, Ainley DG, editors. Great white sharks: The biology of *Carcharodon carcharias*. San Diego: Academic Press. pp. 131–138.

[37] Hoey JJ, Aires-da-Silva A, Turner PA, Syc T, Kohler NE (2005) A review of exploratory longline surveys and biological sampling of sharks from the Sandy Hook, NJ and Narragansett, RI labs: 1961–1991. Southeast Data, Assessment, and Review Data Workshop for Large Coastal Sharks LCS05/06-DW-23.

[38] McCandless CT, Natanson NJ (2010) Standardized catch rates for sandbar and dusky sharks caught during the NEFSC coastal shark bottom longline survey. Southeast Data Assessment and Review 21 Data Workshop for blacknose, sandbar, and dusky shark, SEDAR 21-DW-28.

[39] Hale LF, Gulak SJB, Napier AM, Carlson JK (2011) Characterization of the shark bottom longline fishery: 2010. NOAA Tech Memo NMFS-SEFSC-611.

[40] Lambert D (1992) Zero-inflated Poisson regression, with an application to defects in manufacturing. Technometrics 34 (1): 1–13.

[41] Welsh AH, Cunningham RB, Donnelly CF, Lindenmayer DB (1996) Modelling the abundance of rare species: statistical models for counts with extra zeros. Ecol Model 88: 297–308.

[42] Maunder MN (2001) A general framework for integrating the standardization of catch per unit of effort into stock assessment models. Can J Fish Aquat Sci 58: 795–803.

[43] Maunder MN, Punt AE (2004) Standardizing catch and effort data: a review of recent approaches. Fish Res 70: 141–159.

[44] Conn PB (2010a) Hierarchical analysis of multiple noisy abundance indices. Can J Fish Aquat Sci 67: 108–120.

[45] Conn PB (2010b) Hierarchical analysis of blacknose, sandbar, and dusky shark CPUE indices. Southeast Data Assessment and Review 21 Assessment Workshop for blacknose, sandbar, and dusky shark, SEDAR 21-AP-01.

[46] R Development Core Team (2012). R: A language and environment for statistical computing, reference index version 2.15.2. R Foundation for Statistical Computing, Vienna, Austria. ISBN 3-900051-07-0, URL http://www.R-project.org.

[47] McKinney JA, Hoffmayer ER, Wu W, Fulford R, Hendon JM (2012) Feeding habitat of the whale shark *Rhincodon typus* in the northern Gulf of Mexico determined using species distribution modeling. Mar Ecol Prog Ser 458: 199–211.

[48] Fergusson IK (1996) Distribution and autecology of the white shark in the eastern North Atlantic Ocean and the Mediterranean Sea. In: Klimley AP, Ainley DG, editors. Great white sharks: The biology of *Carcharodon carcharias*. San Diego: Academic Press. pp. 321–345.

[49] Clua E, Seret B (2012) New Caledonia (South Pacific) as a potential tropical wintering ground for the white shark, *Carcharodon carcharias*. In: Domeier ML, editor. Global perspectives on the biology and life history of the great white shark. Boca Raton: CRC Press. pp. 343–353.

[50] Beerkircher LR, Cortes E, Shivji M (2002) Characteristics of shark bycatch observed on pelagic longlines off the southeastern United States, 1992–2000. Mar Fish Rev 64 (4): 40–49.

[51] Springer S (1960) Natural history of the sandbar shark, *Eulamia milberti* . Fish Bull 61 (178): 1–38.

[52] Kohler NE, Casey JG, Turner PA (1998) NMFS cooperative shark tagging program, 1962-1993: An atlas of shark tag and recapture data. Mar Fish Rev 60 (2): 1–87.

[53] Shepherd GR, Moser J, Deuel D, Carlsen P (2006) The migration patterns of bluefish (*Pomatomus saltatrix*) along the Atlantic coast determined from tag recoveries. Fish Bull 104: 559–570.

[54] Weng KC, Boustany AM, Pyle P, Anderson SD, Brown A (2007) Migration and habitat of white sharks (*Carcharodon carcharias*) in the eastern Pacific Ocean. Mar Biol 152: 877–894.

[55] Domeier ML, Nasby-Lucas N (2008) Migration patterns of white sharks *Carcharodon carcharias* tagged at Guadalupe Island, Mexico, and identification of an eastern Pacific shared offshore foraging area. Mar Ecol Prog Ser 370: 221–237.

[56] Bruce BD, Bradford RW (2012) Habitat use and spatial dynamics of juvenile white sharks, *Carcharodon carcharias*, in eastern Australia. In: Domeier ML, editor. Global perspectives on the biology and life history of the great white shark. Boca Raton: CRC Press. pp. 225–254.

[57] Duffy CAJ, Francis MP, Manning MJ, Bonfil R (2012) Regional population connectivity, oceanic habitat, and return migration revealed by satellite tagging of white sharks, *Carcharodon carcharias*, at New Zealand aggregation sites. In: Domeier ML, editor. Global perspectives on the biology and life history of the great white shark. Boca Raton: CRC Press. pp. 301–318.

[58] Goldman KJ (1997) Regulation of body temperature in the white shark, *Carcharodon carcharias* . J Comp Phys B 167: 423–429.

[59] Bonfil R, Meyer MA, Scholl MC, Johnson RL, OBrien S (2005) Transoceanic migration, spatial dynamics, and population linkages of white sharks. Science 310: 100–103. 16210537 10.1126/science.1114898

[60] Francis MP, Duffy CAJ, Bonfil R, Manning MJ (2012) The third dimension: Vertical habitat use by white sharks, *Carcharodon carcharias*, in New Zealand and in oceanic and tropical waters of the southwest Pacific Ocean. In: Domeier ML, editor. Global perspectives on the biology and life history of the great white shark. Boca Raton: CRC Press. pp. 319–342.

[61] Weng KC, OSullivan JB, Lowe CG, Winkler CE, Dewar H, et al (2007) Movements, behavior and habitat preferences of juvenile white sharks *Carcharodon carcharias* in the eastern Pacific. Mar Ecol Prog Ser 338: 211–224.

[62] Tricas TC, McCosker JE (1984) Predatory behavior of the white shark (*Carcharodon carcharias*), with notes on its biology. Proc Cal Acad Sci 43: 221–238.

[63] Curtis TH, Kelly JT, Menard KL, Laroche RK, Jones RE (2006) Observations on the behavior of white sharks scavenging from a whale carcass at Point Reyes, California. Cal Fish Game 92 (3): 113–124.

[64] Wood-Lafond SA (2009) Dynamics of recolonization: A study of the gray seal (*Halichoerus grypus*) in the northeast U.S. Ph.D. dissertation, University of Massachusetts, Boston.

[65] Brodie P, Beck B (1983) Predation by sharks on the gray seal (*Halichoerus grypus*) in Eastern Canada. Can J Fish Aquat Sci 40: 267–271.

[66] Carey FG, Kanwisher JW, Brazier O, Gabrielson G, Casey JG, et al (1982) Temperature and activities of a white shark, *Carcharodon carcharias* . Copeia 1982: 254–260.

[67] Pratt Jr HL, Casey JG, Conklin RB (1982) Observations on large white sharks, *Carcharodon carcharias*, off Long Island, New York. Fish Bull 80 (1): 153–156.

[68] Arnold PW (1972) Predation on harbor porpoise, *Phocoena phocoena*, by a white shark, *Carcharodon carcharias* . J Fish Res Board Can 29: 1213–1214.

[69] Turnbull SD, Dion D (2012) White shark (*Carcharodon carcharias*) attack on a harbor porpoise (*Phocaena phocaena*) in the Bay of Fundy, Canada. Northeast Nat 19 (4): 705–707.

[70] Klimley AP, Anderson SD, Pyle P, Henderson RP (1992) Spatiotemporal patterns of white shark (*Carcharodon carcharias*) predation at the South Farallon Islands, California. Copeia 1992: 680–690.

[71] Strong Jr WR, Murphy RC, Bruce BD, Nelson DR (1992) Movements and associated observations of bait-attracted white sharks, *Carcharodon carcharias*: a preliminary report. Aust J Mar Freshw Res 43: 13–20.

[72] Kock A, ORiain MJ, Mauff K, Meyer M, Kotze D (2013) Residency, habitat use and sexual segregation of white sharks, *Carcharodon carcharias* in False Bay, South Africa. PLoS ONE 8 (1): e55048 10.1371/journal.pone.0055048 23383052 PMC3557240

[73] Adam PJ (2004) *Monachus tropicalis* . Mammal Spec 747: 1–9.

[74] Taylor JK, Mandelman JW, McLellan WA, Moore MJ, Skomal GB (2012) Shark predation on North Atlantic right whales (*Eubalaena glacialis*) in the southeastern United States calving ground. Mar Mam Sci 29 (1): 204–212.

[75] Garrison LP (2007) Defining the North Atlantic right whale calving habitat in the southeastern United States: An application of a habitat model. NOAA Tech Memo NOAA-NMFS-SEFSC-553.

[76] Long DJ, Jones RE (1996) White shark predation and scavenging on cetaceans in the eastern North Pacific Ocean. In: Klimley AP, Ainley DG, editors. Great white sharks: The biology of *Carcharodon carcharias*. San Diego: Academic Press. pp. 293–307.

[77] National Marine Fisheries Service (1993) Fishery management plan for sharks of the Atlantic Ocean. United States Department of Commerce, National Oceanic and Atmospheric Administration, National Marine Fisheries Service, Silver Spring, Maryland.

[78] Dudley SFJ, Simpfendorfer CA (2006) Population status of 14 shark species caught in the protective gillnets off KwaZulu-Natal beaches, South Africa, 1978–2003. Mar Freshw Res 57: 225–240.

[79] Reid DD, Robbins WD, Peddemors VM (2011) Decadal trends in shark catches and effort from the New South Wales, Australia, Shark Meshing Program 1950–2010. Mar Freshw Res 62: 676–693.

[80] Gubili C, Duffy CAJ, Cliff G, Wintner SP, Shivji M (2012) Application of molecular genetics for conservation of the white shark, *Carcharodon carcharias*, L. 1758. In: Domeier ML, editor. Global perspectives on the biology and life history of the great white shark. Boca Raton: CRC Press. pp. 357–380.

[81] Bruce BD, Stevens JD, Malcolm H (2006) Movements and swimming behaviour of white sharks (*Carcharodon carcharias*) in Australian waters. Mar Biol 150 (2): 161–171.

