# Supplementary figures and images for: Seasonal Distribution and Historic Trends in Abundance of White Sharks, Carcharodon carcharias, in the Western North Atlantic Ocean
Source: PLoS One. 2014 Jun 11;9(6):e99240. doi: 10.1371/journal.pone.0099240 (PMC4053410; doi:10.1371/journal.pone.0099240)

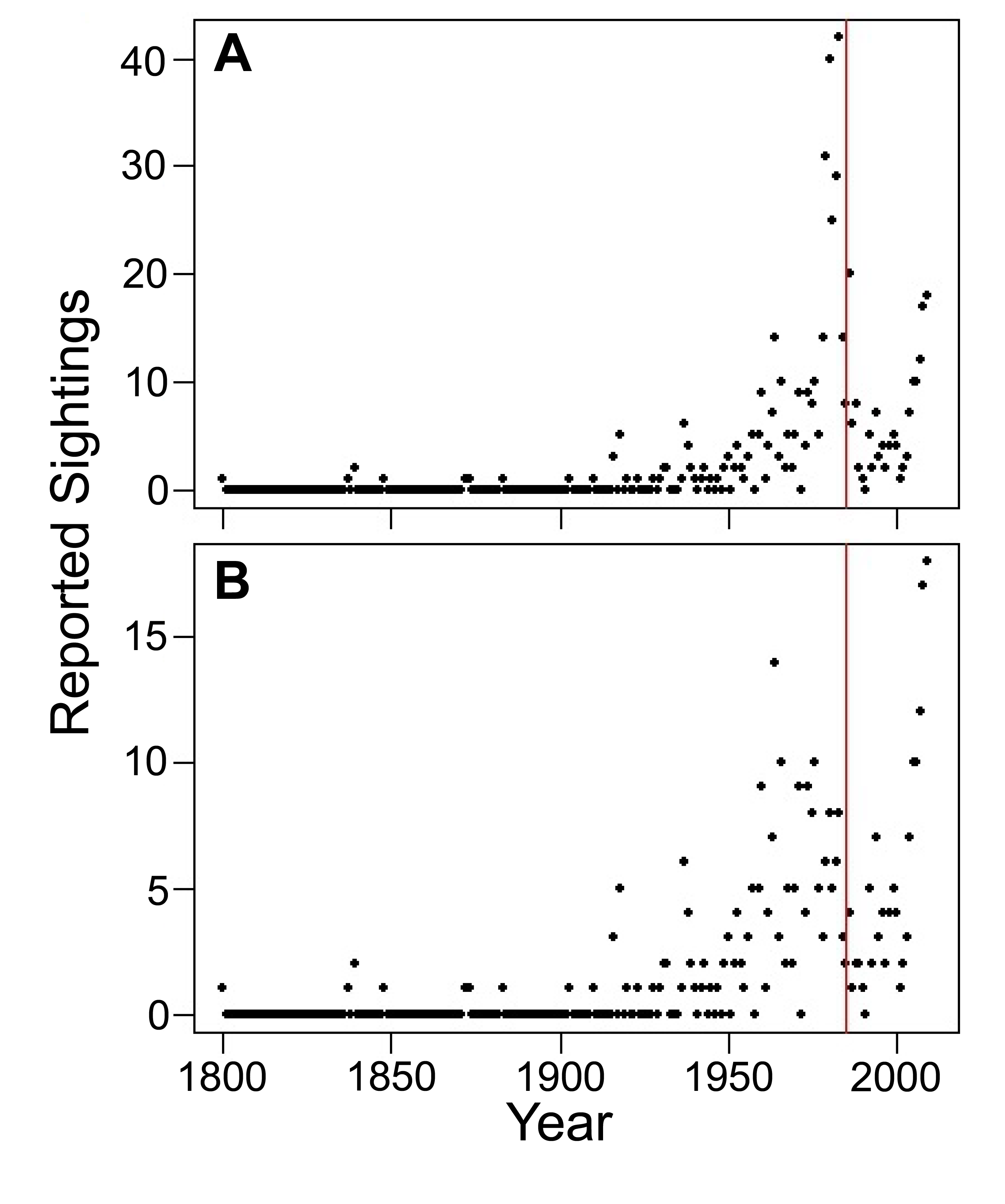

Supplement: Figure S1 — Time series of white shark sightings. (a) Number of annual white shark sightings reported in the NWA from 1800 to 2009, excluding the time series used in the hierarchical analysis and recent directed effort. The vertical red line indicates the year the first comprehensive NWA white shark distribution paper was published [24]. (b) Number of annual white shark sightings used to model trends in abundance, contains an 80% reduction in records leading up to and directly following the Casey and Pratt [24] publication (red line) to account for directed effort during that time. (TIF) [file pone.0099240.s001.tif]

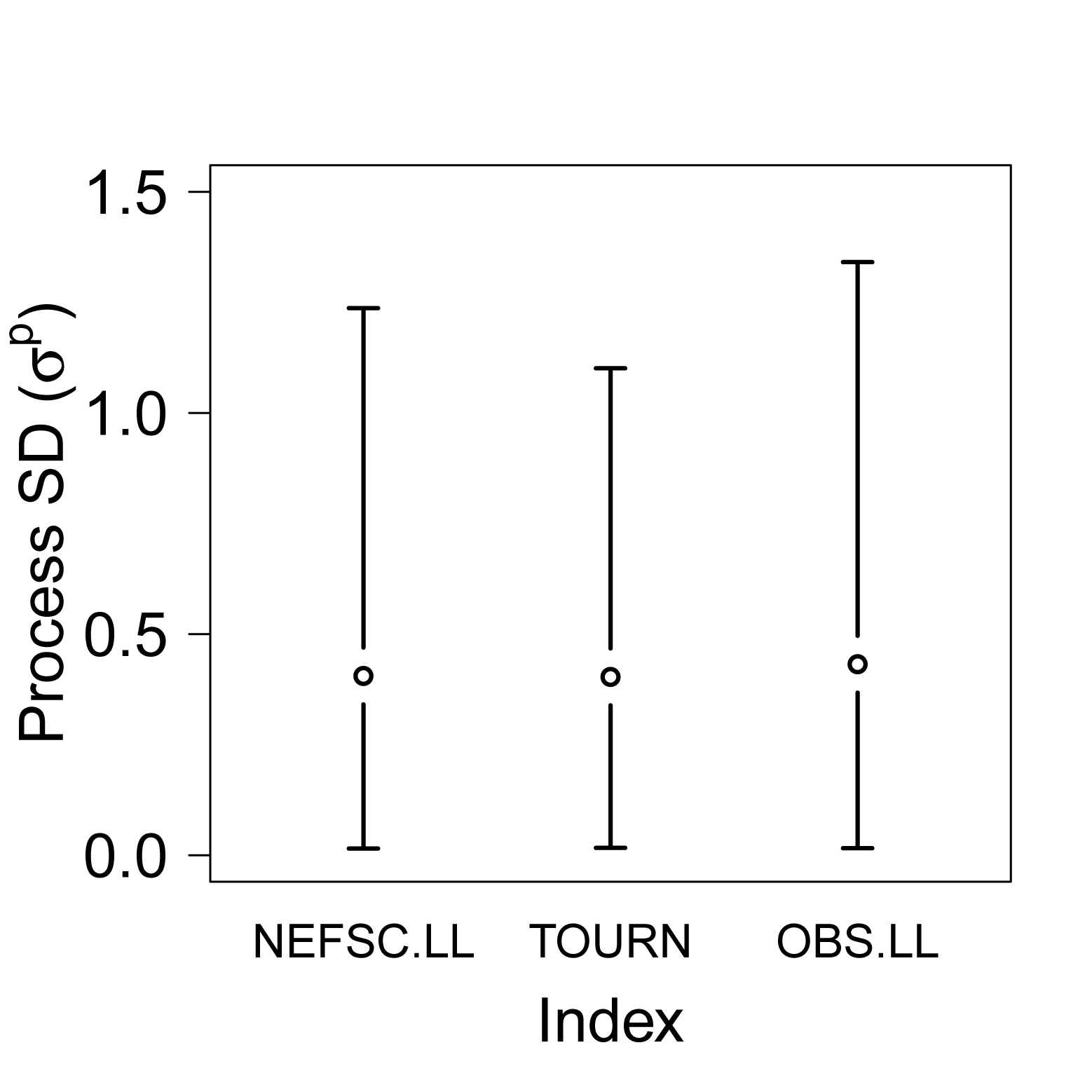

Supplement: Figure S2 — Process errors for white shark relative abundance indices. Posterior means and 95% credible intervals for the standard deviation (SD) of process error for the three indices used in the hierarchical analysis. NEFSC LL = Northeast Fisheries Science Center fishery-independent longline surveys, TOURN = NEFSC tournament database, and OBS LL = observer program of the directed shark longline fishery. (TIF) [file pone.0099240.s002.tif]
